# Alzheimer’s Disease: The Current and Emerging Treatment Approaches

**DOI:** 10.1155/bn/9627699

**Published:** 2025-12-07

**Authors:** Runxuan Pang, Qi Jia, Chen Ma, Tinghai Li, Wenliang Bi, Hongyang Wang, Rongyu Liu, Pengyuan Chen, Eui-Seok Lee, Heng Bo Jiang

**Affiliations:** ^1^ The Conversationalist Club & Department of Dental Digitalization, School of Stomatology, Shandong First Medical University, Jinan, Shandong, China, sdfmu.edu.cn; ^2^ Department and Research Institute of Dental Biomaterials and Bioengineering, Yonsei University College of Dentistry, Seoul, Republic of Korea, yonsei.ac.kr; ^3^ Department of Oral and Maxillofacial Surgery, Korea University Guro Hospital, Seoul, Republic of Korea, kumc.or.kr

**Keywords:** Alzheimer’s disease, amyloid *β*, brain stimulation techniques, gene therapy, pharmacotherapy, stem cell therapy, therapeutic mechanism

## Abstract

Alzheimer’s disease (AD) is a chronic progressive neurodegenerative disease characterized by amyloid *β* (A*β*) plaques and neurofibrillary tangles (NFTs) as its main pathological features. It mainly manifests as cognitive dysfunction, and its pathological process may occur before symptom onset. However, the current drugs and methods for treating AD have unsatisfactory therapeutic outcomes. Therefore, finding a treatment that can inhibit the progression of AD by targeting its pathological features is an urgent need. This review summarizes the current traditional drugs that can delay the progression of AD and new drugs that act on the pathological characteristics of AD and highlights the potential value of related plant extracts. In addition, this review explores the application of different vectors, such as viral vectors and nanoparticles, in gene therapy and drug delivery. These data will provide novel ideas for new drug development and the search for new therapeutic mechanisms.

## 1. Introduction

**Figure 1:**
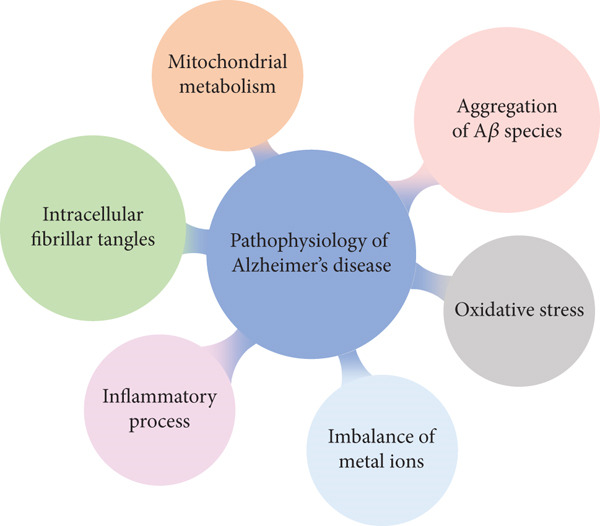
Pathophysiological features of AD. The pathogenesis of AD is unknown; the main hypotheses are the A*β* hypothesis and the tau hypothesis. Moreover, AD is accompanied by various pathological features, such as intracellular fibrillar tangles, oxidative stress, inflammatory processes, and mitochondrial metabolism.

AD is a neurodegenerative disease with progressive cognitive impairment. It is pathologically characterized by cortical atrophy with A*β* deposits, NFTs, and the loss of several functional neurons. However, the pathogenesis of AD is unknown and involves various pathological features (Figure 1) [1, 2]. Of the 55 million people living with dementia worldwide, 75%–80% are estimated to have AD [3]. However, so far, no drugs have been developed to cure or reverse the pathological process of AD. The pathological features persist for several years before the onset of clinical symptoms. Therefore, in addition to the treatment of AD, its prevention and early diagnosis should be emphasized [4].

In the traditional drug therapy for AD, therapeutic drugs are mainly divided into two categories: N‐methyl‐d‐aspartate glutamate receptor (NMDAR) antagonists that can maintain the normal physiological function of the NMDAR and acetylcholinesterase inhibitors (AChEIs) that can maintain the normal level of acetylcholine (ACh) [5]. However, traditional drugs associated with adverse symptoms, such as liver toxicity, can only be used to treat oxidative stress and neuroinflammation associated with AD and have no obvious effect on eliminating A*β* plaques and tau pathologies [6]. If traditional drugs exhibit poor efficacy, several newly developed drugs can be used to eliminate A*β* plaques. These drugs exhibit innovative mechanisms of action, which are summarized in this paper. Aducanumab, for example, is the first and currently the only treatment option to eliminate A*β* plaques. Sodium oligomannate, piracetam, and masitinib slow the progression of AD act by promoting energy conversion, targeting the microbiota–gut–brain axis, and targeting the neuroimmune system, respectively [7].

Some plant extracts are also effective against AD and have fewer side effects than traditional drugs, potentially providing some new research directions. However, few studies have been conducted on plant extracts, and their active substances and therapeutic mechanisms remain unclear. The blood–brain barrier (BBB) can limit the therapeutic efficacy of drugs, while nanoparticles (NPs) and viral vectors can improve the permeability and bioavailability of drugs in the BBB. Moreover, NPs and viral vectors can be used to treat AD by transporting various genes, such as PGC‐1*α* and ApoE2 genes. However, their use is limited by the sustained action time of the genes transported by the carrier in the body. Stem cell therapy and brain stimulation therapy are still relatively immature. Stem cell therapy has long been considered a safe and effective method to replace damaged or dead neurons in the brain with cholinergic neurons of stem cell origin. However, there is a lack of clinical data on stem cell therapy. Moreover, transplantation is limited to intravenous, intracerebral, and cerebrospinal fluid injections [8]. For invasive brain stimulation (IBS), the main method is deep brain stimulation (DBS). But its equipment such as pulser and electrode have obvious shortcomings, less innovation, and brain stimulation surgery is more complicated and less safe. In comparison, noninvasive brain stimulation (NIBS) might be a better option.

This review integrates in vivo and in vitro experimental data to clearly analyze the mechanisms of action, efficacy, and safety of drugs, comprehensively presenting the multiple values of classic drugs in AD treatment, such as reducing A*β* levels and inhibiting immune cell activation. Meanwhile, it focuses on analyzing the novel mechanisms of action of new drugs, including inhibiting microglia and mast cell activation and targeting the brain–gut axis to regulate gut microbiota for AD treatment, to highlight their research value. In terms of pathological mechanisms, besides systematically sorting out classical theories such as A*β* liposomes and tau protein hypothesis, this review also elaborates on two emerging mechanisms, neuroinflammation and metal ion damage, to comprehensively interpret the classical and cutting‐edge mechanisms of the disease pathology. The therapeutic strategies cover traditional and new drugs, herbal therapies such as curcumin and huperzine A, stem cell therapies, as well as diversified delivery systems like NPs and viral vectors, providing readers with a comprehensive interpretation from pathological mechanisms to therapeutic approaches.

## 2. Pharmacotherapy

Toll‐like receptors (TLRs), Wnt signaling pathways, and mast cells, among others, play a crucial role in regulating the pathological progress of AD through different mechanisms; they can be targeted by certain drugs to relieve AD. A combination of the TLR and its ligands activates the innate immune system and plays a vital role in the adaptive immune response. TLR 2 activation promotes neurogenesis in the hippocampus, while TLR 2 deficiency causes cognitive dysfunction. TLR 3 and TLR 4 regulate neuronal proliferation and differentiation, TLR 5 loss causes memory decline, and TLR 3/7/8 activation prevents neurons from connecting to harmful substances and increases dendritic spine density [9]. In addition, TLR signaling can be beneficial to patients with AD through the uptake of A*β* plaques. However, similar to microglia, it can also induce inflammation, which is harmful to patients. TLR 2 and TLR 4 agonists may avoid neuronal death caused by glutamate‐induced excitotoxicity [10].

Wnt glycoprotein signaling can be activated through three major cellular pathways: a canonical Wnt signaling pathway (the Wnt/*β*‐catenin signaling pathway) and two noncanonical pathways (the Wnt/Ca^2+^ pathway and the planar cell polarity pathway). The Wnt signaling pathway is essential for various systems related to organism growth and development and can harmonize various cellular processes, including cell differentiation and proliferation; cell senescence and death; and the proliferation, differentiation, and migration of adult stem cells. The Wnt signaling pathway plays a particularly important role in patients with AD by regulating neurogenesis; the formation of glutamatergic synapses; and the proliferation, differentiation, and migration of neural precursors. The loss of Wnt signaling can directly affect synapses, causing synaptic vulnerability, and neurons are more susceptible to A*β*‐induced apoptosis [11–13].

The hyperactivation of mast cells is associated with the abnormal aggregation of A*β* plaques in the brain of patients with AD. Astrocytes produce and release cytokines, tumor necrosis factor‐*α* (TNF‐*α*), and chemokines, which can activate microglia. Microglial activation can trigger mast cell degranulation and induce the release of various mediators, including histamine, resulting in the aggregation of A*β* plaques; this in turn can promote microglial activation and aggravate neuroinflammation [14].

The hallmark signs of astrocyte reactivity appear in the early stages of cognitive decline. The early appearance of astrocyte reactivity in patients with AD may provide a key upstream mechanism for many complex and highly interrelated processes in AD, including neuroinflammation, synaptic dysfunction, cerebral vascular pathology, and hypometabolism. Oral administration is the most commonly used mode of drug delivery, mainly in the form of tablets and capsules. Rivastigmine, donepezil, huperzine A, galantamine, and memantine, which are used in the treatment of AD, are mainly oral preparations and this type of drug delivery is mainly suitable for patients with AD who need to take the drugs for a long period of time. However, their absorption and clinical efficacy are easily affected by the digestive system, and gastrointestinal administration is difficult to achieve brain targeting. Patients with acute attacks of AD are unable to take medication, so injections are mostly used for acute attacks of AD. The injections that have been marketed for the treatment of AD include galantamine injection and piracetam injection, etc. However, their production costs are high and they are prone to adverse effects. There are also alternative therapies for transdermal delivery, including patches, emulsions, gels, and microneedles, which are noninvasive route of administration and safer than injections. However, only rivastigmine transdermal patches and donepezil microneedles are currently on the market and are susceptible to skin keratinization. In addition to generic liquid formulations, microemulsions, liposomes, polymeric NPs, and in situ gels, which are administered through the nasal cavity, are rapidly developing in recent years, which are faster, more efficiently absorbed and more bioavailable than conventional delivery modalities [15].

### 2.1. AChEIs

In patients with AD, an abnormal increase in acetylcholinesterase (AChE) activity can increase the velocity of ACh catabolism, resulting in a lower level of ACh in the brain and consequently affecting the transmission of nerve impulses in the central nervous system. Part of this enzyme acts as a promoter and is involved in the formation of A*β* plaques and NFTs. It also facilitates the aggregation of A*β* peptide fragments by forming complexes with fibrils, thereby accelerating the progression of AD [16]. At present, the most commonly used drugs to treat AD are AChEIs, such as tacrine, donepezil, galantamine, and rivastigmine. However, tacrine has been withdrawn from the market because of liver toxicity. All four drugs can be used to treat mild to moderate AD, while higher doses of donepezil (10 and 23 mg/tablet) can be used to treat moderate to severe AD. Notably, drug derivatives designed on the basis of the drug’s own fundamental properties and exo‐binding ability have better efficacy against AD, and play an important role in optimizing drug design and development strategies [17].

#### 2.1.1. Donepezil

In vivo experiments on mice with AD revealed that the accumulation of A*β*_1-40_ and A*β*_1-42_ in the mitochondria was significantly reduced after donepezil treatment. The symptoms of mitochondrial swelling were also significantly reduced. In vitro experimental T‐maze behavior tests in AD mouse models revealed that vector‐treated mice exhibited lower accuracy in T‐mazes than wild‐type controls; donepezil treatment significantly alleviated this loss of working memory [18]. In short‐term human trials, patients with AD treated with donepezil showed significant improvements in cognitive ability [19]. Donepezil has a relative bioavailability of 100% after oral administration. Moreover, it has no dose‐limiting hepatotoxicity and has a longer half‐life (70–80 h). Therefore, high doses of donepezil can be used to treat patients with moderate to severe AD [20, 21].

#### 2.1.2. Galantamine

Similar to donepezil, galantamine is a selective and rapidly reversible AChEI that acts as an allosteric enhancer of nicotine receptors. It thereby enhances neurotransmitter release and alleviates cognitive impairment, as the loss of nicotine receptors exacerbates the severity of cognitive impairment. Moreover, galantamine inhibits lipopolysaccharide (LPS) activity, which exposes cytokines (TNF‐*α*, interleukin‐1*β*, and interleukin‐6) and inflammatory signaling molecules (NF‐*κ*B and p65) in the hippocampus, thereby preventing LPS from affecting the activation of microglia and astrocytes to combat neuroinflammation [19]. In addition to fighting inflammation, galantamine reduces the amount of A*β* in the brain by boosting microglial function. It is clinically hypothesized that galantamine prevents or delays the development of AD if administered in the pre‐A*β* plaque phase [22].

#### 2.1.3. Rivastigmine

Unlike other AChEIs, rivastigmine has a lower binding force to proteins but higher selectivity. It preferentially inhibits haplotype (G1) AChE over tetramer (G4) AChE. During the progression of AD, G1 AChE accumulates and plays an important role in the hydrolysis of the neurotransmitter ACh [19]. Rivastigmine esterifies the ester site of AChE through the carbamate portion. This carbamate esterification lasts for up to 10 h and persists even after rivastigmine leaves the AChE ester site and is hydrolyzed. AChE, which is esterified by carbamate, loses its ability to hydrolyze ACh. Compared with other AChEIs, rivastigmine has fewer peripheral side effects. For example, its elimination half‐life is only 2 h and it is directly transformed into inactive metabolites at the site of action, without requiring liver metabolism. However, the inhibitory effect of rivastigmine on AChE is dose‐dependent [23].

#### 2.1.4. Neostigmine

Neostigmine is a carbamate derivative that reversibly inhibits AChE and promotes ACh production at the ACh delivery site. It reversibly inhibits the active site of AChE and the function of AChE through the carbamylation of serine and is finally decomposed by AChE and metabolized by liver microsomal enzymes. However, neostigmine does not cross the BBB and has a low gastrointestinal absorption rate when consumed orally [23]. Nanotechnology can improve the bioavailability of drugs in the CNS. The use of NPs could promote the passage of neostigmine through the BBB and its eventual release into brain tissues via directional control, which may help combat AD [24].

### 2.2. NMDAR Antagonist

In patients with AD, abnormal increases in glutamate levels are often beyond the control of the body’s glutamate buffer system. Glutamate that aggregates near extracellular receptors can produce excitatory signals and cause cytotoxicity, resulting in the overactivation of the NMDAR and causing excessive calcium influx, which can eventually damage or kill neurons. Memantine, the only NMDAR antagonist on the market, can play an antagonistic role by blocking the overactivation of the NMDAR. It can act as an inhibitor to prevent the damage caused by excessive calcium influx even at high glutamate levels. Memantine can also enhance autophagy to remove swollen or dysfunctional mitochondria from neurons resulting from excessive calcium influx in order to resist neurodegeneration characterized by abnormal mitochondrial accumulation in patients [17, 22, 25]. In addition, memantine can block oxidative stress induced by A*β* oligomers (ABOs) and relieve oxidative stress by reducing A*β* levels and neuroinflammation, thereby alleviating symptoms of AD [26, 27].

Interestingly, the effect of a patient’s psychological state at the time of treatment may warrant further investigation. In a human trial involving patients with moderate AD, the memantine group exhibited greater deterioration of AD after 24 weeks of treatment, while the memantine and music group exhibited an improvement, indicating that music therapy can improve the psychological behavior of patients with AD to a better extent than drug therapy alone, thereby enhancing the efficacy of drug therapy [28]. As a drug combination therapy, memantine can also be used in combination with AChEIs because the two drugs have complementary modes of action. For example, the combination of memantine and galantamine can have a better curative effect on patients. It can exhibit the same cascade of neuroprotective effects and dual antioxidant effects, which may be enough to counteract the complex cascade of inflammation, cell damage, and tissue damage caused by the redox state [2].

### 2.3. Emerging Therapeutic Agents

#### 2.3.1. Monoclonal Antibodies

Aducanumab is the first agent to treat AD by reducing the amount of A*β* plaques in the brain. It is an anti‐A*β* monoclonal antibody that rapidly targets ABOs and insoluble fibrils in the brain across the BBB at low concentrations and activates microglia to remove A*β* plaques because of its univalent affinity [7]. Aducanumab binds to a linear epitope on amino acids 3–7 of the amyloid peptide chain and exhibits greater affinity and selectivity (more than 10,000 times) to A*β* plaques than A*β* monomers, which is an important mechanism by which it reduces A*β* plaques in neurons. Similar to aducanumab, zagotenemab (MAPT monoclonal antibody) is an anti‐tau antibody that can bind and neutralize tau to treat AD. However, it is only effective in the initial stage of AD [29]. Lecanemab is an anti‐amyloid monoclonal antibody that targets soluble A*β* protofibrils to treat patients with early AD. In the CLARITY AD study based on lecanemab, a significant improvement was noted in the cognitive performance of participants who received intravenous lecanemab after 18 months of treatment. However, lecanemab treatment has the adverse effect of causing amyloid‐related imaging abnormalities, which is a common side effect in patients receiving immunotherapy with A*β* antibodies [30, 31]. Two other monoclonal antibodies are expected to be approved in the future: donanemab and gantenerumab [32].

#### 2.3.2. Piracetam

Piracetam is a cyclic derivative of the neurotransmitter gamma‐aminobutyrate (GABA), which improves cognitive function without causing any sedation or stimulation and promotes ATP production by shifting the mitochondria from a fission/fusion equilibrium state to a fusion state [33, 34]. In an LPS‐induced mouse model of AD, LPS decreased glutathione (GSH) levels and increased reactive oxygen species (ROS) levels. After treatment with pyracetam, GSH and ROS levels returned to normal, indicating that pyracetam can induce brain cells to enhance antioxidant capacity and significantly inhibit ROS levels. In addition, the LPS‐induced overproduction of inflammatory cytokines, such as interleukin‐1*β*, was significantly reduced after treatment with piracetam [35, 36].

#### 2.3.3. Sodium Oligomannate

**Figure 2:**
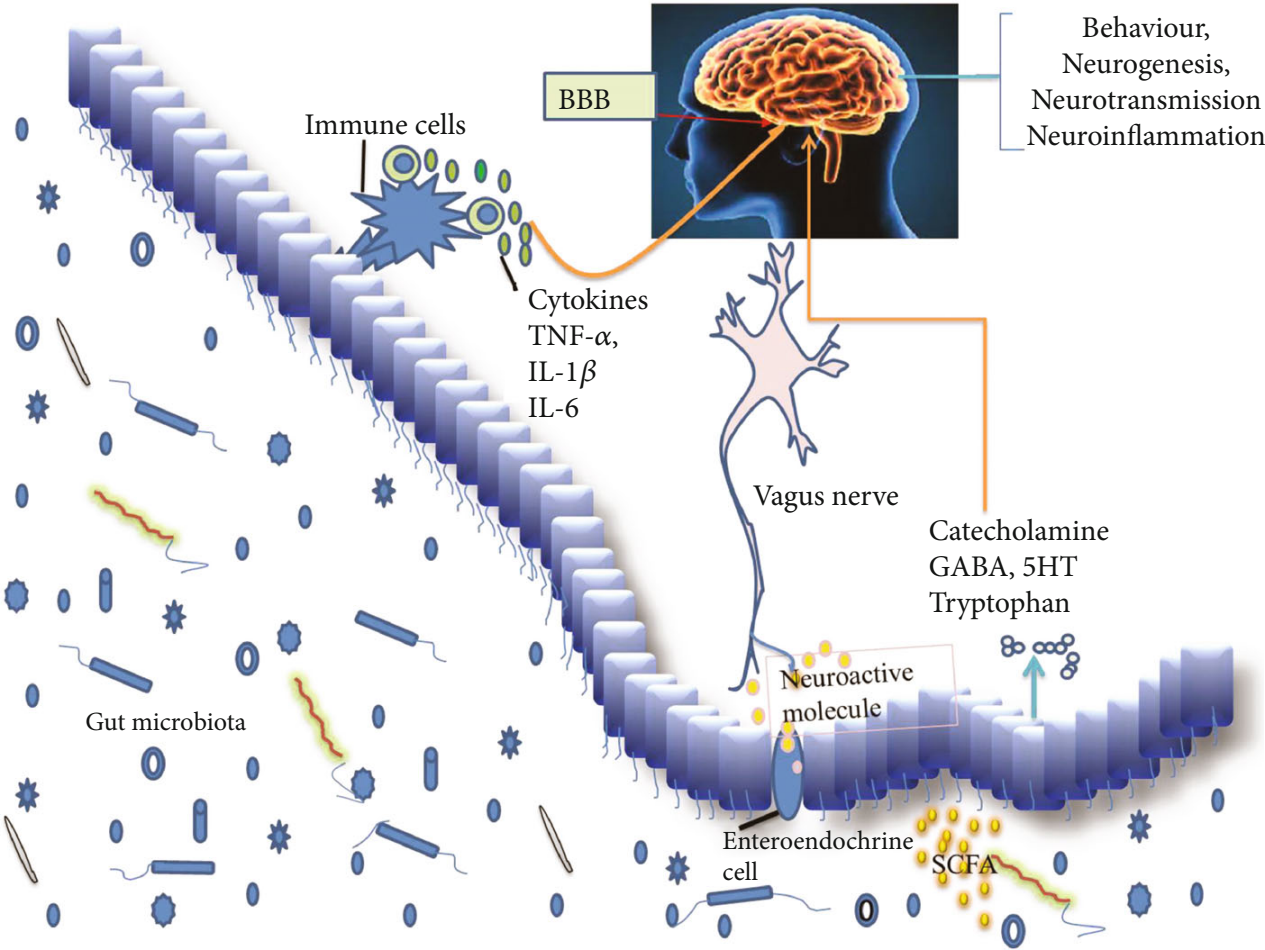
Bidirectional communication between the gut microbiota and the gut–brain axis. Gut microbe‐derived molecules can cross the BBB to stimulate the production of neurotransmitters in the host and trigger inflammatory responses in the gut and brain.

In healthy individuals, the gut microbiota produces beneficial metabolites, such as short‐chain fatty acids (SCFAs), to maintain the integrity of the BBB. However, in patients with AD, an imbalance in the intestinal microbiota leads to a decrease in beneficial metabolites and triggers neuroinflammation and other reactions, thereby causing BBB damage and further promoting the deposition of A*β* plaques (Figure 2). Sodium oligomannate can indirectly regulate intestinal flora in the microbiota–gut–brain axis, relieve neuroinflammation and A*β* plaque deposition caused by intestinal flora imbalance, and directly inhibit the formation of A*β* fibrils to treat mild to moderate AD [37–39].

#### 2.3.4. Masitinib

Unlike other drugs, masitinib targets the brain’s innate immune system. It is an oral tyrosine kinase inhibitor used to treat mild to moderate AD. It remodels the neuronal microenvironment by inhibiting mast cell and microglial activity and transforms the neuroimmune system from a neurotoxic state to a neuroprotective state, exhibiting neuroprotective effects in patients with AD. Masitinib can inhibit the hyperactivation of mast cells by inhibiting tyrosine kinase activity. It can also improve cognitive impairment by inhibiting macrophage colony‐stimulating factor receptor type I (MCSFR1) to target microglia [40, 41]. These findings suggest that masitinib can alleviate cognitive impairment caused by mast cells and microglia, which is a pathological manifestation in patients with AD [42, 43].

### 2.4. Herbal Remedies

#### 2.4.1. Curcumin

Curcumin, a natural compound of the herb turmeric, reduces neurotoxicity and thereby improves cognition in patients with AD by inhibiting central AChE activity and preventing tau hyperphosphorylation. Moreover, it treats AD by regulating metal homeostasis and lipid levels in the body [44].

Patients with AD often have elevated levels of copper, zinc, and other metal ions in the brain. In particular, they have elevated serum copper levels. Curcumin can reduce serum copper levels in these patients and thus be effective in treating AD [45]. Hyperlipidemia is a vital morbific factor in AD; the lipid levels in patients with AD are 10% higher than those in healthy individuals. In a mouse model of obesity, Ding et al. demonstrated that curcumin could improve lipid levels and reduce lipid synthesis by lowering insulin sensitivity, reflecting its potential to prevent AD [46]. In recent studies, molecular hybridization has been utilized to combine the pharmacophoric groups present in curcumin with those present in other AD drugs, resulting in a series of novel compounds that can enhance therapeutic efficacy through multiple mechanisms. Curcumin is generally delivered into the body through NPs to achieve the therapeutic effect of treating AD [47].

#### 2.4.2. Bacopa monnieri

*Bacopa monnieri* is a medicinal plant that improves memory loss, anxiety, poor cognitive performance, and poor concentration [48]. Mitochondrial damage in neurons is considered a risk factor for neurodegenerative diseases, such as AD. Mitochondria in the brain of patients with AD are more susceptible to ROS because of active neuronal metabolism. The metabolic function of the mitochondria is also correlated with A*β* levels. In contrast, studies have demonstrated that *Bacopa monnieri* can reduce plaque‐related oxidative stress by restoring mitochondrial metabolic function and may also reduce A*β* plaque deposition, thereby acting as a neuroprotective agent [49]. In an experiment using a *Cryptobacterium hidradenum* model, mitochondrial damage accumulated within experimentally cultured neurons could be treated using hexane extracts of *Bacopa monnieri* [50]. Chaudhari et al. assessed patients with AD after 1 year of treatment with *Bacopa monnieri* and donepezil and found that both had essentially the same therapeutic effect; however, the use of *Bacopa monnieri* was safer and had fewer side effects than the use of donepezil. In their preclinical trial, Chaudhari et al. did not note any serious neurological or hematological complications associated with the use of *Bacopa monnieri* for the treatment of AD, which also suggests the safety and feasibility of using *Bacopa monnieri* for treating AD. Current studies have assisted in the treatment of AD by extracting triterpenoid saponins from *Bacopa monnieri* [51–53].

#### 2.4.3. Anthocyanin

Anthocyanin are emerging compounds used for the treatment of AD in recent years. It is a red or purple pigment mainly found in plants. In particular, black beans, grapes, and blackcurrants are rich in anthocyanins [54]. Natural plant‐derived anthocyanins have antioxidant, anti‐inflammatory, anticancer, and anti‐neurodegenerative effects. Anthocyanins may prevent numerous chronic neurodegenerative diseases, including AD, and various cardiovascular diseases. They also have the potential to be used as a dietary supplement in patients with AD [55–57].

In patients with AD, anthocyanins may act through multiple pathways. Studies have shown that anthocyanins may intervene in the pathogenesis of AD through the interaction of *π*–*π* with hydrophobic amino acid residues present in A*β* plaques. They may also act together with cyanidin‐3‐glucoside (C3G) to prevent cerebral ischemia and A*β* plaque‐induced mitochondrial degeneration [56, 58]. Moreover, anthocyanins may improve the immunity of patients with AD. The activity of the nuclear factor kappa light chain enhancer is stronger in the brain of patients with AD, causing the immune system to function poorly. In contrast, a study revealed that anthocyanins could downregulate the p65 protein in rats, thereby blocking the activation and expression of the nuclear factor kappa light chain enhancer [59]. Notably, the efficacy of anthocyanins in alleviating oxidative stress, cholinergic dysfunction, tau hyperphosphorylation, and cognitive deficits has been demonstrated in a combination of AD rodent models [60]. An experimental study involving APP/PS1 mice revealed that the endogenous neuroprotective antioxidant PI3K/Akt/Nrf2 pathway activated by anthocyanins has great potential in preventing apoptosis and neurodegeneration related to AD [56].

#### 2.4.4. Lycopene (LYC)

LYC, an aliphatic hydrocarbon carotenoid present in ripe tomatoes, has been found to exhibit quenching activity and anti‐inflammatory effects of free radicals in AD model experiments and could serve as a latent compound for treating neurodegenerative diseases [61]. Moreover, LYC is effective in reducing the levels of oxidative stress markers and could be used to improve LYC deficiency, which is common in the elderly and patients with AD [62].

In mouse experiments, 8 weeks of LYC treatment (5 mg/kg) reduced tau hyperphosphorylation at AD‐related sites by reversing the increase in malondialdehyde (MDA) levels and decrease in GSH peroxidase (GSH‐Px) levels in the serum of tau transgenic mice with a P301 L mutation. Moreover, long‐term LYC treatment (5 mg/kg for 21 days) activated cysteine‐3, which could improve neuronal damage in the hippocampus and reduce A*β*_1-42_ aggregation in the lateral ventricles [63]. Another *in vitro* study in male Wistar rats revealed that LYC administered via the intracerebroventricular (ICV) route using a stereotactic apparatus attenuated mitochondrial damage in primary cultured rat cortical neurons in a dose‐dependent manner [64].

#### 2.4.5. Huperzine A

Huperzine A is an unsaturated sesquiterpenoid alkaloid compound that effectively crosses the BBB. It is a mixed competitive, reversible, and selective inhibitor of AChE. In a mouse study, Zhang et al. found that huperzine A exhibited a stronger inhibitory effect than commonly used AChEIs, such as galantamine, donepezil, tacrine, and carboplatin. Friedli et al. also found that huperzine A exhibited a good neuroprotective effect in an AD model (APPswe/PS1 mice) [65, 66].

Huperzine A promotes ACh accumulation by inhibiting AChE, thereby enhancing ACh signaling for neuroprotection. The neuroprotective mechanism of ACh signaling may be as follows: First, ACh signaling may regulate neural progenitor cells and thus exert neuroprotective effects through the mitogen‐activated protein kinase/extracellular signal‐regulated kinase (MAPK/ERK) pathway, which is activated by the action of lithophane–methyl isoforms [67]. Second, cholinergic signaling increases neurotrophic factor expression, enhances synaptic activity, antagonizes the NMDAR, and regulates ROS production, thereby facilitating neuronal survival [66]. Finally, ACh signaling increases the expression of two proteins, *α*7nAChR and *α*4*β*2nACh, to exert anti‐inflammatory effects [68]. In addition to exerting neuroprotective effects through enhanced ACh signaling, stigmasterol may exert neuroprotective effects by reducing A*β* plaque deposition in the subcellular hippocampus and modulating A*β* peptide toxicity [69, 70]. The therapeutic effect of stigmasterol on AD was also confirmed by Xu et al., who noted improved cognition and memory in 58% of patients with AD who ingested 0.2 mg of stigmasterol.

Huperzine A may improve A*β* plaque aggregation and cognitive deficits in patients with AD by activating Wnt signaling, which is involved in interneuronal connections and has non‐cholinergic neuroprotective effects. The modulation of Wnt signaling may be the most promising treatment for AD; however, the molecular association between huperzine A and the Wnt signaling pathway for neuronal function recovery remains unclear [66].

## 3. Gene Therapy

### 3.1. Viral Vectors

The advantage of using viral vectors for gene therapy is that they can provide higher gene transfer efficiency both in vivo and in vitro. Moreover, some vectors are capable of sustained expression of the target gene [71]. The main viral vectors used for gene therapy in patients with AD are adenoviral vectors (ADVs), adeno‐associated viruses (AAVs), and lentiviruses [72].

#### 3.1.1. ADVs and AAVs

ADVs cannot insert the genes they carry into the host genome, resulting in relatively transient transgene expression; however, ADVs are well tolerated and have minimal side effects and a high safety profile. So far, few studies have used ADVs as gene vectors to treat neurodegenerative diseases [73]. Recent developments in ADVs suggest that ADVs can elicit an immune response in the brain. Intramuscular injection of ADVs encoding neurotrophic factor‐3 in a mouse model of a motor neuron disease mutation could increase the lifespan of mice by exerting substantial therapeutic effects [73].

AAV is a broadly infectious gene vector with low immunogenicity [74]. The genes that AAVs can carry for treating AD include those encoding soluble amyloid precursor protein *α* (sAPP*α*), neprilysin (NEP), endothelin‐converting enzyme 1 (ECE1), apolipoprotein E2 (ApoE2), and transcription factor EB (TFEB), among others. sAPP*α* plays a crucial role in the treatment of AD, affecting synaptic growth and plasticity; the products of the hydrolysis of this protein can also have a range of effects on neurons and the brain. In vitro and in vivo studies on sAPP*α* have suggested that sAPP*α* reduces memory dysfunction in patients with AD [75, 76]. NEP is an important neuropeptidase and amyloid‐degrading enzyme. Although NEP can degrade plaques, the rapid elimination of A*β* plaques leads to a loss of A*β* plaques and the rapid generation of new A*β* plaques; therefore, a single delivery of NEP is not effective in eliminating all plaques in the brain. However, delivering NEP into the brain via an AAV vehicle before many plaques have formed can help achieve sustained delivery, resulting in better plaque elimination [77]. When delivered in vivo, AAV capsids are less immunogenic than ADVs, have a lower toxicity potential, are not pathogenic at the doses required for transduction induction, and have lower side effects. AAVs are therefore known as the safest viral vectors for gene therapy and provide good transgene expression [78, 79].

#### 3.1.2. Lentiviruses

Lentiviruses are commonly used to study host nondividing cells, such as those of the nervous and cardiac systems. They are now widely used in gene therapy for AD [80]. Although both lentiviruses and AAVs can integrate their genomes into the host cell genome for longer‐term transgene expression, lentiviruses can host a larger gene capacity [79]. They can carry several genes, such as those encoding CREB, PGC‐1*α*, NEP, ApoE2, PP2A, and Parkin. Of these, the CREB gene is an important molecular target gene for learning and memory. A reduction in CREB signaling may be one of the mechanisms underlying cognitive impairment in AD [75, 81].

### 3.2. NPs

NP‐mediated drug delivery systems improve the solubility and bioavailability of drugs. The main NPs currently available for use in AD therapy are liposomal NPs, chitosan NPs, synthetic polymeric NPs, gold NPs, magnetic NPs, carbon nanotubes, carbon dots, and curcumin NPs [82]. NPs are mainly administered orally and intravenously into the body and accumulate around the blood vessels of the brain to function [83]. In general, the challenges of low efficiency and poor precision are inherent when using NP‐mediated drug delivery systems for gene therapy; these concerns become even more pronounced when these systems are applied to AD drug delivery [72].

#### 3.2.1. Liposomal NPs

At present, the most widely used nonviral gene vectors are liposomal NP vectors [73]. Liposomal vectors offer many advantages over viral vectors, such as greater safety, the ability to transfect larger amounts of genetic material, and lower toxicity [71]. The main genes that can be transported by liposomal NPs are those encoding mouse‐ApoE (mApoE), *β*‐sheet breaker peptide H102 (H102), and methoxy‐XO4 (XO4), among others [82]. The pathology of AD is characterized by A*β* plaque aggregation and tau hyperphosphorylation. H102 directly prevents and reverses the misfolding and aggregation of A*β* in conformational disorders. It also plays a crucial role in the treatment of AD and has a significant effect on the expression of several related proteins, including tau, inflammatory factors, and apoptotic factors [84].

#### 3.2.2. Chitosan NPs

The main advantages of chitosan NPs include better biocompatibility and higher biodegradability. The main substances that can be transported by chitosan NPs are tacrine, growth hormone (GH), and piperine, among others [82]. Piperine is the main alkaloid isolated from black pepper. It has been shown to have the potential for treating neurodegenerative diseases, such as AD, in animal models [85].

#### 3.2.3. Polymer‐Based NPs

There are many drugs that can be used to synthesize polymer NPs and here we have chosen curcumin, which is more studied, as a representative. Curcumin enhances gene expression for neuronal differentiation by activating the Wnt/*β*‐catenin‐linked protein pathway and may help treat neurodegenerative diseases, such as AD, by enhancing the brain’s self‐healing mechanisms [86]. Despite these impressive attributes, curcumin faces substantial challenges in clinical application because of its poor bioavailability, rapid metabolism, and limited ability to penetrate the blood–brain barrier. Pluronic F127 NPs (FCur NPs) encapsulated with curcumin promote blood circulation and enhance BBB permeability to help curcumin effectively cross the BBB for intracranial migration. Moreover, experiments have revealed that curcumin NPs exhibit a stronger binding capacity to A*β* plaques than Congo red. Therefore, FCur NPs have the potential to be a theranostic agent for AD [87].

#### 3.2.4. Magnetic NPs

The main substances that can currently be transported by magnetic NPs are quercetin and the triphenylphosphine cation triphenylphosphine (TPP) [82]. Quercetin inhibits A*β* plaque production and tau phosphorylation as well as AChE; however, the limitation associated with quercetin use is its low bioavailability. An experiment on Wistar rats revealed that quercetin delivered into the brain using quercetin‐conjugated superparamagnetic iron oxide NP (QT‐SPION) carriers had a better neuroprotective effect than free quercetin, resulting in a better therapeutic effect in patients with AD [88].

## 4. Stem Cell Therapy

### 4.1. Neural Stem Cells (NSCs)

NSCs are self‐renewing pluripotent stem cells that are widely involved in the maintenance and repair of human brain homeostasis. NSCs show multieffect immanence and can differentiate into various cell types, such as astrocytes, oligodendrocytes, and neurons [89]. In a transgenic NSE/APPsw AD mouse model carrying the mutated human APP gene, the degree of tau phosphorylation and content of A*β*_1-42_ in the brain of mice were significantly reduced within 5 weeks after the injection of NSCs. Gliosis was also relieved. From Week 7, NSCs began to migrate and differentiate to replenish the depleted NSC pool in the brain; they mainly differentiated into immature neurons [90].

NSCs not only replace dead and dying neurons but also create a favorable microenvironment for the CNS and secrete therapeutic gene products, such as nerve growth factor (NGF), brain‐derived neurotrophic factor (BDNF), and glial cell‐derived neurotrophic factor (GDNF), which promote endogenous neurogenesis and synaptogenesis [8]. NSCs can also reduce abnormal tau aggregation by regulating various proteins involved in neurogenesis and long‐term potentiation (LTP), significantly improving memory. N‐acetylaspartic acid and glutamate levels were increased in patients after NSC transplantation, suggesting that NSCs not only increase the number of neurons but also reconstruct functional neuronal circuits [91]. In addition, although NSCs reduce the expression of TLR, which inhibits the secretion of pro‐inflammatory factors and slows down neuroinflammation, they do not affect the content of A*β* plaques [8].

At present, NSCs can be obtained through direct neural tissue extraction, in vitro expansion using basic fibroblast growth factors (FGFs), and induced multipotent stem cell (iPSC) and embryonic stem cell (ESC) differentiation. However, the differentiation process is too complicated. Moreover, the phenotypes of NSCs obtained using different methods are different [8, 92]. Compared with other stem cells, NSCs have a lower risk of tumor development and immunogenicity. Furthermore, NSCs are most similar to human brain neurons, resulting in a higher safety guarantee during transplantation [89].

### 4.2. Mesenchymal Stem Cells (MSCs)

MSCs are pluripotent stem cells with multidirectional differentiation ability and high self‐renewal ability. They can differentiate into non‐mesenchymal lineages. MSCs can phagocytose deposited A*β* plaques by differentiating into microglia or activating endogenous microglia. In addition, they can play an anti‐inflammatory role by reducing the release of pro‐inflammatory factors and upregulating anti‐inflammatory factors, such as interleukin‐10. Moreover, studies have shown that the anti‐inflammatory ability and immunomodulatory properties of MSCs can be enhanced through 3D culture technology [93–95].

Human umbilical cord MSCs (hUC‐MSCs) not only have the characteristics of MSCs but also have no ethical problems and tumorigenicity. They can also be easily purified and separated. Moreover, hUC‐MSCs can promote hippocampal neurogenesis by secreting GDNF, NGF, BDNF, and FGF‐2 and activating the Wnt signaling pathway through parasecretory effects [96]. In a senescence‐accelerated mouse prone 8 (SAMP8) model of AD, hUC‐MSCs were found to secrete hepatocyte growth factor (HGF) to improve the structure and function of damaged neurons and promote synaptic plasticity. They could also target and downregulate hyperphosphorylated tau at the cellular level. In addition, hUC‐MSCs could target A*β* plaques through migration and differentiation pathways, which in turn could differentiate and activate microglia and enhance the autophagy function of cells. Autophagy is a physiological cellular stress response that removes harmful and redundant substances from cells through the function of lysosomes. Moreover, microautophagy in the brain plays an important role in maintaining the normal function of synapses [97]. This promotes the elimination of A*β* plaques, reduces the damage of A*β* plaques to neurons, and improves the survival rate of neurons [91].

Human menstrual blood‐derived stem cells (MenSCs) have the advantages of being easy to obtain, having no ethical problems, and having a higher proliferation rate. In mouse experiments, AD mice transplanted with MenSCs showed significantly improved spatial learning and memory and significantly enhanced activities of several A*β*‐degrading enzymes. Compared with the control mice, the redox reaction in the brain of AD mice transplanted with autologous bone marrow‐derived MSCs (BM‐MSCs) was markedly reduced, suggesting that BM‐MSCs slow down the progression of AD by controlling oxidative stress [93].

Compared with other stem cells, MSCs have the advantages of high safety performance, wide sources, easy operation, and immunomodulatory properties. However, they still have the disadvantages of poor diffusion after injection and incomplete in vitro culture technology. Given the immunomodulatory properties of MSCs, the chances of viral and fungal infections increase after transplantation. Inadequate cell survival, paracrine effects, and limited homing ability after crossing the BBB also limit the therapeutic benefits of MSCs [92, 94].

### 4.3. iPSCs

iPSCs use viral vectors to insert embryonic genes or specific gene products into somatic cells in order to reprogram them back into a “stem‐like” state. Early iPSC treatment regimens completely rely on the reprogramming of patients’ somatic cells. Reprogramming is mainly accomplished using a mixture of four transcription/reprogramming factors (TFs/RFs): c‐MYC, KLF4, SOX2, and OCT4 [92, 98].

However, this process is relatively complex, the reprogramming efficiency is low, and the induction of iPSCs into neurons for a long time can lead to PSEN1/2 mutations, which can affect the normal morphology and function of neurons [99]. Because of the insertion of embryonic genes, iPSCs may also form teratomas as ESCs do. Moreover, iPSCs exhibit interdonor differences and intercell heterogeneity. Compared with other stem cells, iPSCs have the outstanding advantages of no immune rejection caused by autologous transplantation and no histocompatibility [92].

**Figure 3:**
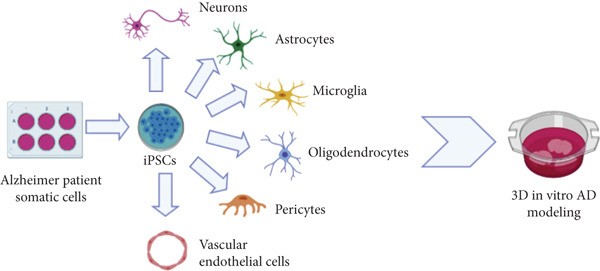
AD model. iPSCs reprogrammed with patients’ somatic cells can differentiate into a wide range of neural cells for in vitro modeling, which can be used to assess drug efficacy and study interactions between different cell types.

iPSCs can continuously differentiate into various cell types affected by AD. Moreover, they can differentiate into quite mature neurons both structurally and functionally, which can be used to form active synaptic circuits. The multilineage differentiation ability of iPSCs also provides a model platform for the study of AD (Figure 3); this platform can be used not only to evaluate potential drugs for the treatment of AD but also to simulate the interaction between nerve cells *in vivo* for studying the cascade reaction caused by A*β* plaques and hyperphosphorylated tau in patients with AD [100, 101]. The most recent treatment of iPSCs involves the transdifferentiation of somatic intermediates into inducible neural precursor colonies (iNPCs), which have a comparable differentiation ability to NSCs but are more likely to differentiate into cholinergic neurons. The transdifferentiation process is more convenient, shorter, and cheaper than the reprogramming process [89].

### 4.4. ESCs

ESCs are special stem cells that originate in the inner cell mass of blastocysts and have totipotency and high self‐renewal ability. So far, ESCs have been mainly differentiated into astrocytes and neuron‐like cells to treat AD [102]. The degeneration of basal forebrain cholinergic neurons (BFCNs) and loss of synapses are associated with cognitive impairment in patients with AD. In an AD mouse experiment, ESCs transplanted into mice with AD were effectively differentiated into BFCN progenitor cells, which mainly differentiated into mature cholinergic neurons, thereby improving the function of the endogenous cholinergic system. Moreover, the experimental mice showed significant improvements in cognitive function [103, 104]. Compared with other stem cells, ESCs exhibit greater immune rejection. As ESCs are obtained from the blastula, ethical issues persist. Although ESCs exhibit totipotency, they may also differentiate into tumor cells and form teratomas. Furthermore, undifferentiated ESCs may cause uncontrolled cell division. Differentiated ESCs may also produce different phenotypes [92].

## 5. Brain Stimulation Therapy

Brain stimulation therapy can regulate cognitive function in patients with AD and can be divided into IBS and NIBS. At present, the main brain stimulation technique used for the treatment of AD is TMS, a type of NIBS.

The therapeutic efficiency of transcranial magnetic stimulation (TMS), a type of NIBS, in patients with AD is worthy of further research. TMS operates by generating short, high intensity magnetic pulses (up to 300 *μ*s and 2.5 Tesla, respectively) through a copper wire coil applied to the scalp [105]. This technique delivers rapidly changing electric current to specific brain regions, primarily in the superficial layers of the cortex. Cortical neuron modulation and dosage are determined by the stimulation intensity and by the individual’s motor‐evoked potential threshold [106]. Notably, mice treated with high‐frequency rTMS exhibited better outcomes. Choung et al. demonstrated that early administration of rTMS (round coil, 1 or 20 Hz at 1.26 T) improved cognitive behavioral deficits induced by A*β*1–42 injection in mice by activating the dopaminergic system and upregulating neurogenic signaling. Additionally, enhanced recovery effects were observed with high‐frequency rTMS, as proven in their in vivo experiments [107]. Lee and colleagues conducted a study involving 26 patients with mild or moderate AD. They targeted six brain regions with high‐frequency (10 Hz) rTMS: bilateral DLPFC, Broca’s area, Wernicke’s area, and bilateral somatosensory cortices (R‐pSAC and L‐pSAC, respectively). The participants were randomly assigned to two groups: the first group received 30 rTMS sessions over a period of 6 weeks, while the second control group received sham rTMS. The treatment group showed significant improvements in ADAS‐Cog scores after the six‐week intervention, as well as in the MMSE and CGIC scores. Subgroup analysis revealed that the effects were more pronounced in the mild AD group, particularly in the domains of memory and language [108].

In addition, Fontaine et al. reported that patients with mild cognitive decline exhibited relatively stable cognitive performance and improved metabolism of the medial temporal lobe after 12 months of DBS treatment [109]. However, the field of brain stimulation therapy for treating AD is still relatively immature and in the experimental stage [105]. A 1‐year phase I trial conducted to study the effects of DBS treatment in six patients with early‐stage AD revealed improvements in memory and cognitive decline after 12 months of DBS treatment. As the phase I trial proved that DBS can improve memory and cognitive function in patients with AD, the researchers further recruited 42 patients with mild AD for a phase II study. However, the effect of DBS treatment was not obvious in the phase II trial. Elderly patients with AD who received DBS treatment even experienced significant deterioration compared with patients who did not receive DBS treatment [110].

## 6. Conclusion

Given the complexity of the brain and the fact that lesions in the brain of patients with AD may persist for several years before the onset of obvious clinical symptoms, the results of current treatments for AD are largely unsatisfactory. Therefore, in addition to the direct treatment of patients with medication or other methods, the early detection and treatment of AD are crucial. Currently approved drugs for treating AD are mainly limited to AChEIs and NMDAR antagonists. Although several new drugs hold promising prospects, these drugs still require further research and larger‐scale trials. The treatment of AD using plant extracts is a possible option; however, their active substances and therapeutic mechanisms remain unclear. Moreover, experimental evidence regarding their feasibility is lacking. At present, the clinical application of stem cell therapy is limited by issues related to ethical aspects, tumorigenicity, transplantation methods, post‐transplantation mechanisms, and immune rejection. However, stem cell therapy still has great potential and prospects not only for the direct treatment of AD but also for the construction of AD models to simulate the in vivo environment of patients with AD and assess the therapeutic effects of drugs. In the gene therapy section, we have also compared the advantages and disadvantages of different vectors to select more suitable vectors and provide suggestions for the construction of new vectors. Different delivery methods of drugs and genes can produce different effects; therefore, the construction of new vectors and pathways should also be emphasized. If AD cannot be reversed by treatment, raising awareness and finding ways to prevent the disease may be as important as treating it.

## Conflicts of Interest

The authors declare no conflicts of interest.

## Author Contributions

Runxuan Pang, Qi Jia, and Chen Ma contributed equally to this work.

## Funding

This works was supported by Korea University Guro Hospital (KOREA RESEARCH‐DRIVEN HOSPITAL) and grant funded by Korea University Medicine (No. K2515191).

## Data Availability

No data was used for the research described in the article.
